# Supplementary material for: Sex Difference on Neurological Outcomes and Post-Cardiac Arrest Care in Out-of-Hospital Cardiac Arrest Patients Treated with Targeted Temperature Management: Post-Hoc Study of a Prospective, Multicenter, Observational Cohort Study
Source: J Clin Med. 2023 Aug 14;12(16):5297. doi: 10.3390/jcm12165297 (PMC10455960; doi:10.3390/jcm12165297)
Supplement: Supplementary file 1 [file jcm-12-05297-s001.zip › jcm-2543691-supplementary.pdf]

Table S1. Associating factors for undergoing early cardiac diagnostic and therapeutic interventions in arrest patients with presumed cardiac etiology.

	Early coronary angiography*				Early PCI**				Early echocardiography				ECMO			
	Univariate		Multivariable		Univariate		Multivariable		Univariate		Multivariable		Univariate		Multivariable	
	OR (95% CI)	P	OR (95% CI)	P	OR (95% CI)	P	OR (95% CI)	P	OR (95% CI)	P	OR (95% CI)	P	OR (95% CI)	P	OR (95% CI)	P
Men sex	1.61 (1.16-2.24)	0.005	1.18 (0.82-1.69)	0.373	1.88 (1.10-3.21)	0.021	1.25 (0.56-2.80)	0.584	1.16 (0.89-1.51)	0.278	1.02 (0.77-1.35)	0.888	3.33 (1.50-7.38)	0.003	2.51 (1.11-5.66)	0.027
Age, per year	1.00 (0.99-1.00)	0.273			1.02 (1.01-1.04)	0.004	1.01 (0.99-1.04)	0.363	1.00 (0.99-1.01)	0.915			0.99 (0.97-1.00)	0.057		
mCI 0	Ref															
mCI 1	0.89 (0.63-1.25)	0.502	0.97 (0.66-1.42)	0.868	0.92 (0.56-1.52)	0.755			0.96 (0.71-1.29)	0.768			0.88 (0.46-1.68)	0.696		
mCI 2	0.61 (0.39-0.96)	0.034	0.69 (0.42-1.15)	0.154	0.66 (0.32-1.37)	0.263			0.96 (0.64-1.42)	0.820			0.83 (0.34-2.00)	0.677		
mCI 3	0.45 (0.26-0.77)	0.004	0.76 (0.41-1.39)	0.365	1.45 (0.61-3.44)	0.402			1.13 (0.75-1.69)	0.563			0.78 (0.30-2.01)	0.599		
Witnessed	1.07 (0.75-1.52)	<0.001	0.83 (0.56-0.23)	0.351	2.03 (1.18-3.48)	0.010	1.24 (0.54-2.85)	0.615	1.65 (1.26-2.17)	<0.001	1.34 (1.00-1.80)	0.049	1.40 (0.78-2.54)	0.263		
Bystander CPR	1.11 (0.83-0.47)	0.100			0.95 (0.62-1.46)	0.817			1.17 (0.92-1.50)	0.209			1.15 (0.68-1.96)	0.605		
Shockable rhythm	2.44 (1.83-3.25)	<0.001	1.92 (1.37-2.67)	<0.001	2.46 (1.57-3.85)	<0.001	3.64 (1.83-7.27)	<0.001	1.69 (1.33-2.16)	<0.001	1.16 (0.85-1.56)	0.351	3.03 (1.80-5.15)	<0.001	1.35 (0.75-2.41)	0.317
Downtime, min†	1.00 (0.99-1.01)	0.701			0.99 (0.98-1.01)	0.285			0.99 (0.99-1.00)	0.166			1.01 (1.00-1.03)	0.104		
Cardiac etiology	NA				NA				2.07 (1.59-2.68)	<0.001	1.64 (1.19-2.26)	0.002	9.52 (3.43-26.37)	<0.001	5.51 (1.85-16.38)	0.002
Motor grade > 2	1.86 (1.32-2.63)	<0.001	1.55 (1.03-2.34)	0.037	0.82 (0.51-1.32)	0.414			1.42 (1.03-1.96)	0.031	1.09 (0.92-1.82)	0.138	1.08 (0.54-2.17)	0.824		
Brainstem reflex after ROSC	1.77 (1.27-2.47)	0.001	1.16 (0.78-1.74)	0.467	1.40 (0.87-2.23)	0.163			1.22 (0.89-1.67)	0.215			1.71 (0.94-3.11)	0.080		
STEMI or LBBB	4.99 (3.48-7.15)	<0.001	4.76 (3.25-6.97)	<0.001	5.62 (3.53-8.95)	<0.001	3.81 (1.95-7.47)	<0.001	1.70 (1.24-2.33)	0.001	1.29 (0.92-1.82)	0.138	5.44 (3.21-9.20)	<0.001	3.24 (1.85-5.67)	<0.001
Shock on TTM	1.23 (0.93-1.62)	0.144			0.89 (0.59-1.34)	0.585			0.77 (0.61-0.98)	0.032	0.81 (0.63-1.04)	0.101	2.22 (1.28-3.85)	0.005	2.64 (1.50-4.67)	0.001
Stenosis ≥ 50%†	NA				197.64 (47.44-823.44)	<0.001	171.26 (39.71-738.51)	<0.001	NA				NA			

*In presumed cardiac etiology arrest (n=829)
**In patients underwent early CAG (n=368)
† Time from arrest to ROSC, min
Abbreviations: mCI, modified Charlson comorbidity index; CPR, cardiopulmonary resuscitation; GCS, Glasgow coma scale; STEMI, ST elevation myocardial infarction; LBBB, left bundle branch block; TTM, targeted temperature management; PCI; percutaneous coronary intervention, ECMO; extracorporeal membrane oxygenation.
